# Functional Analysis of Two Zinc (Zn) Transporters (*ZIP3* and *ZIP8*) Promoters and Their Distinct Response to *MTF1* and *RREB1* in the Regulation of Zn Metabolism

**DOI:** 10.3390/ijms21176135

**Published:** 2020-08-26

**Authors:** Shu-Wei Chen, Kun Wu, Wu-Hong Lv, Fang Chen, Chang-Chun Song, Zhi Luo

**Affiliations:** 1 Key Laboratory of Freshwater Animal Breeding, Ministry of Agriculture, Fishery College, Huazhong Agricultural University, Wuhan 430070, China; chenshuwei@webmail.hzau.edu.cn (S.-W.C.); pervcy@webmail.hzau.edu.cn (K.W.); lvwuhong@webmail.hzau.edu.cn (W.-H.L.); chenfang95@webmail.hzau.edu.cn (F.C.); songchangchun@webmail.hzau.edu.cn (C.-C.S.); 2 Laboratory for Marine Fisheries Science and Food Production Processes, Qingdao National Laboratory for Marine Science and Technology, 1 Wenhai Road, Qingdao 266237, China

**Keywords:** fish, Zn homeostasis, *ZIP* transporter, transcriptional regulation, Zn toxicity

## Abstract

*ZIP* (zinc-regulated transporters, iron-regulated transporter-like protein) family plays an important role in organism Zn balance. This research identified the promoter regions of *ZIP3* and *ZIP8*, two members of *ZIP* family, from a freshwater teleost yellow catfish *Pelteobagrus fulvidraco*, characterized the binding sequences of the metal-responsive transcription factor-1 (*MTF-1*) and Ras responsive element binding protein 1 (*RREB1*) on their promoter regions. The present study cloned and obtained the 2027 bp of *ZIP3* promoter and 1664 bp of ZIP8 promoter, and predicted several key elements on their promoters, such as the binding sites of *CREB* (cAMP-response element binding protein), *KLF4* (Kruppel like factor 4), *MTF-1* and *RREB1*. The sequence deletion from −361 bp to −895 bp down-regulated the luciferase activity of *ZIP3* promoter, and the deletion from −897 bp to −1664 bp down-regulated the luciferase activity of *ZIP8* promoter. Within different deletion plasmids, the relative luciferase activities of *ZIP3* and *ZIP8* promoters changes to Zn incubation in a Zn concentration-dependent manner. The site mutagenesis and EMSA (electrophoretic mobility shift assay) found that the −1327 bp/−1343 bp *MTF-1* binding site and the −248 bp/−267 bp *RREB1* binding site on the ZIP3 promoter, and the −1543 bp/−1557 bp *MTF-1* binding site on the *ZIP8* promoter are functional sites. Low Zn increased the binding capability between *MTF-1* and its responsive site on the *ZIP3* promoter, and high Zn increased the transcriptional activation *ZIP3* by *RREB1*; Zn also promoted the binding ability between *MTF-1* and its responsive element on the *ZIP8* promoter. This study provides the first direct evidence for the response elements of *MTF-1* and *RREB1* on *ZIP3* and *MTF-1* on *ZIP8* to Zn, which are very important for the evaluation of Zn nutrition and toxicity in vertebrates.

## 1. Introduction

Zn is a trace element essential for all the organisms because of its important roles in many important physiological processes [1,2]. Zn deficiency will cause adverse physiological effects, particularly during the period of rapid growth [2]. Therefore, organisms possess effective systems to absorb Zn from the environment. Zn uptake is mediated by Zn transporters of the *ZIP* (zinc-regulated transporters, iron-regulated transporter-like protein) family, which increase the cytoplasmic Zn concentration by transporting Zn from the extracellular space or organelles into cytoplasma [3,4]. Among these members, *ZIP3* and *ZIP8* were widely expressed in many tissues and involved in Zn acquisition by many cells of the body [4,5]. However, *ZIP3* and *ZIP8* possess many differences in the tissue distribution, expression, subcellular localization and regulation. These differences have important implications for their functions in maintaining Zn homeostasis. However, little is known about the regulation and function of *ZIP3* and *ZIP8* in fish.

In eukaryotic organisms, transcription initiation regulates the gene expression. Promoters have many cis-acting elements and can be bound by transcriptional factors, which regulate the expression of genes at the transcriptional level. Therefore, as the first step for demonstrating the regulatory mechanism of Zn transporters, it is very important to investigate the structure and function of the promoters. In addition, metal-regulatory transcription factor-1 (*MTF1*) and ras-responsive element-binding protein 1 (*RREB1*) are two important Zn-finger (Cys2His2) transcription factors and function as sensors of intracellular Zn by binding to their corresponding responsive elements, and thereby regulates cellular adaptation to Zn [6,7,8]. At present, the responsive elements of *MTF-1* and *RREB1* have been identified in the promoter regions of several Zn transporters [6,8], but not in *ZIP3* and *ZIP8*. Moreover, the role of Zn in regulating *ZIP3* and *ZIP8* remains unclear.

The yellow catfish (*Pelteobagrus fulvidraco*) is an omnivorous freshwater teleost that is widely cultured in several Asian countries for their excellent fillets and high economic value. In this study, the promoter regions of *ZIP3* and *ZIP8* were identified in yellow catfish, and the binding sites of *MTF-1* and *RREB1* in the promoter regions were studied. This study provides an innovative insight into the mechanism by which Zn regulates the transcriptional activities of *ZIP3* and *ZIP8*, which are very important for the evaluation of Zn nutrition and toxicity in vertebrates.

## 2. Results

### 2.1. Cloning and Sequence Analysis of the ZIP3 ((Zinc-Regulated Transporters, Iron-Regulated Transporter-Like Protein)) and ZIP8 Promoters

**Figure 1:**
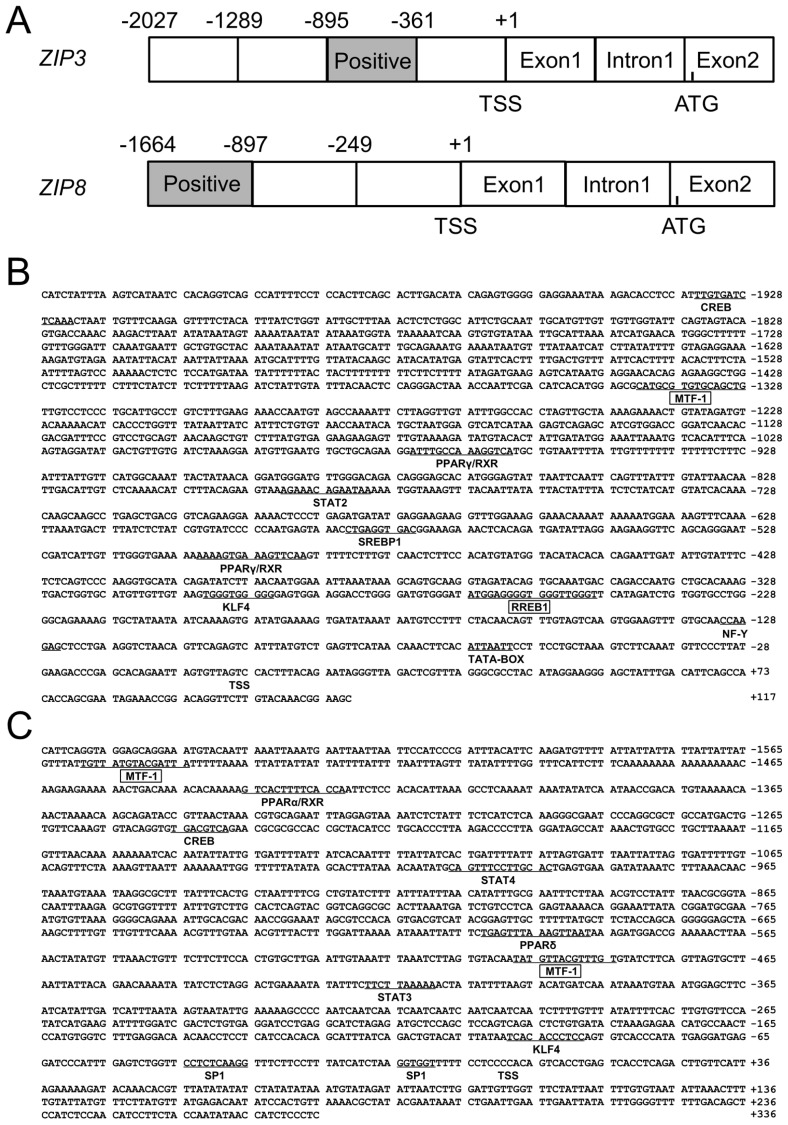
(**A**) The schematic diagram of *ZIP3* and *ZIP8* gene structures. Positive: the region that positively regulated the promoter activity. TSS: transcription start site. ATG: translation initiation site; (**B**) Nucleotide sequence of yellow catfish *ZIP3* promoter. Numbers are relative to the transcription start site (+1). The putative transcription factor binding sites are underlined. The highlighted sequences show putative transcription factor binding sites of *MTF-1* and *RREB1*; (**C**) Nucleotide sequence of yellow catfish ZIP8 promoter. Numbers are relative to the transcription start site (+1). The putative transcription factor binding sites are underlined. The highlighted sequences show putative transcription factor binding sites of *MTF-1*.

The 2027 bp of *ZIP3* promoter and 1664 bp of *ZIP8* promoter were successfully cloned (Figure 1A), and the first nucleotide of 5′ cDNA of *ZIP3* and *ZIP8* were designated as +1. On the *ZIP3* promoter, several core promoter elements, such as a TATA-box (TBP, −60 bp/−67 bp) and a CCAAT-box (nuclear transcription factor Y, NF-Y, −124 bp/−131 bp) were predicted (Figure 1B); a cluster of transcription factor binding sites were also predicted, including *CREB* (−1922 bp/−1935 bp), *STAT2* (signal transducer and activator of transcription 2) (−780 bp/−793 bp), *SREBP1* (sterol-regulatory element binding proteins 1) (−574 bp/−584 bp), *KLF4* (−294 bp/−304 bp) and *PPARγ* (peroxisome proliferators-activated receptors γ) (−489 bp/−505 bp and −960 bp/−975 bp), *MTF-1* (−1327 bp/−1343 bp) and *RREB1* (−248 bp to −267 bp) (Figure 1B). On the *ZIP8* promoter, this study discovered two GC-boxes (Sp1, −8 bp/−14 bp and −34 bp/−44 bp), the binding sites of two *MTF-1* (−483 bp/−497 bp and −1543 bp/−1557 bp) on its core region, *CREB* (−1237 bp/−1245 bp), *STAT3* (signal transducer and activator of transcription 3) (−408 bp/−418 bp), *STAT4* (signal transducer and activator of transcription 4) (−992 bp/−1006 bp), *KLF4* (−87 bp/−98 bp), *PPARα* (peroxisome proliferators-activated receptors α) (at −1421 bp/−1435 bp) and *PPARδ* (peroxisome proliferators-activated receptors δ) (at −586 bp/−602 bp) on its core region (Figure 1C).

### 2.2. Analysis of the 5′-Sequence Deletion of the ZIP3 and ZIP8 Promoters

**Figure 2:**
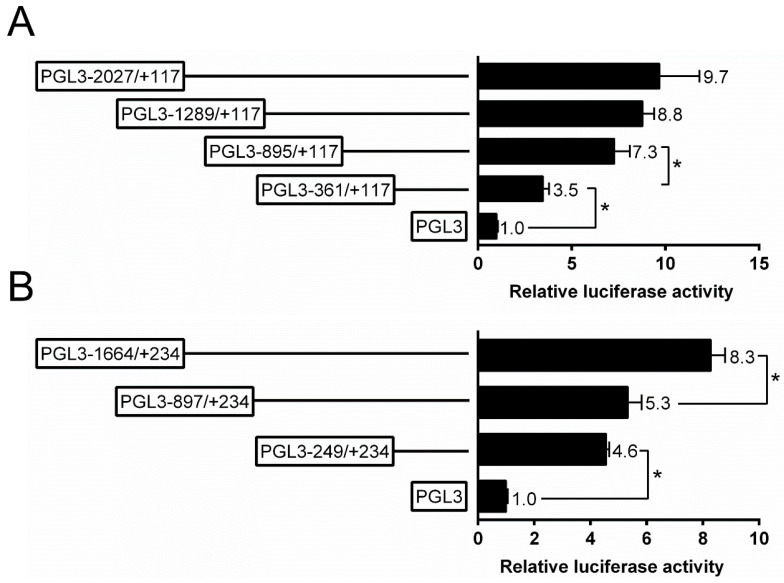
5′ unidirectional deletion assays of the promoter regions of *ZIP3* (**A**) and *ZIP8* (**B**) of yellow catfish. Values mean the ratio of activities of Firefly to Renilla luciferase, normalized to the control plasmid, and are shown as mean ± standard error of mean (SEM) (*n* = 3). Asterisk (*) means significant differences between two groups (*p* < 0.05).

The present study randomly generated the plasmids of different sizes and selected four plasmids for the analysis of 5′-sequence deletion. For *ZIP3* promoter, the sequence deletion from −361 bp to −895 bp down-regulated the luciferase activity significantly, and the sequence deletion from −895 bp to −2027 bp did not significantly affect the luciferase activity (Figure 2A). For the *ZIP8* promoter, the subsequent absence from +234 to −897 bp did not affect the luciferase activity, whereas the deletion from −897 bp to −1664 bp down-regulated the luciferase activity (Figure 2B).

**Figure 3:**
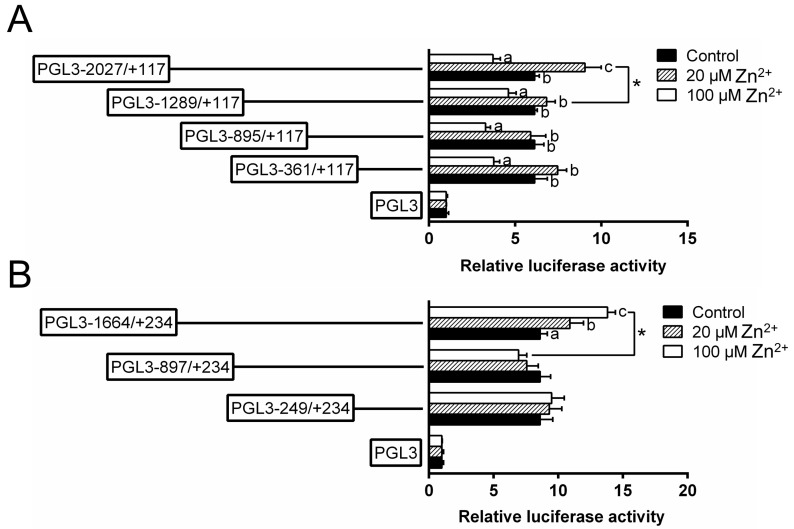
5′ unidirectional deletion assays for promoter regions of *ZIP3* (**A**) and *ZIP8* (**B**) after Zn incubation. Values showed the ratio of activities of Firefly to Renilla luciferase, normalized to the control, and were presented as mean ± SEM (*n* = 3). Asterisk (*) indicates significant differences between different 5′ unidirectional deletion plasmids under the same treatment (*p* < 0.05). Different letters (a, b and c) indicate significant differences among different treatments in the same plasmid (*p* < 0.05).

To investigate the responses of these promoters to Zn, 20 μmol/L (low Zn) and 100 μmol/L Zn^2+^ (high Zn) were used to incubate the HEK293T cells and conducted the 5′-sequence deletion assay (Figure 3). For the *ZIP3* promoter, within the deletion plasmids of −2027 bp/+117 bp, the relative luciferase activities were the lowest in the high Zn group and the highest in the low Zn group; for other plasmids, the luciferase activities in the high Zn group were significantly lower than those in other two groups. For the *ZIP8* promoter, within the plasmid of −1664 bp/+234 bp, the luciferase activities increased with increasing Zn concentrations; however, for other plasmids, Zn incubation did not significantly affect the luciferase activities. Meantime, when the sequence was deleted from −1664 bp to −897 bp, high Zn significantly reduced the luciferase activities.

### 2.3. Site-Mutation Analysis of MTF-1 (Metal-Responsive Transcription Factor-1) and RREB1 (Ras Responsive Element Binding Protein 1) Binding Sites on the ZIP3 and ZIP8 Promoters

**Figure 4:**
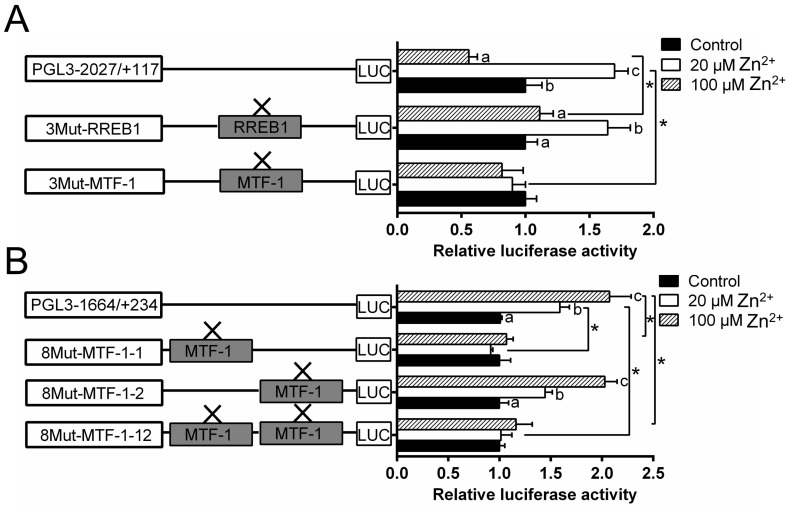
Assays of predicted *MTF-1* and *RREB1* binding sites of yellow catfish *ZIP3* and *ZIP8* promoters after site-directed mutagenesis. (**A**) Site mutagenesis of *MTF-1* on pGl3-*ZIP3*-2027 vector and *RREB1* on pGl3-*ZIP3*-2027 vector; (**B**) site mutagenesis of *MTF-1* on pGl3-*ZIP8*-1664 vector. Values mean the ratio of activities of Firefly to *Renilla* luciferase, normalized to the control. Results were presented as mean ± SEM (*n* = 3). Asterisk (*) indicates significant differences between different 5′ unidirectional deletion plasmids under the same treatment (*p* < 0.05). Different letters (a, b and c) indicate significant differences between different treatments in the same plasmid (*p* < 0.05).

Since *MTF-1* and *RREB1* mediated the regulation of Zn homeostasis, it is important to determine if the observed changes in gene expression are directly related to their regulation. Thus, this study performed the analysis of site mutation in the *ZIP3* and *ZIP8* promoters that may possess *MTF-1* and *RREB1* binding sites. For the *ZIP3* promoter, the mutation of the −1327/−1343 *MTF-1* binding site (3Mut-*MTF-1*) influenced the Zn-induced variation of the luciferase activity, indicating that this site acted as a functional one for the *ZIP3* transcriptional response to Zn incubation; the mutation of the −248/−267 *RREB1* binding site (3Mut-*RREB1*) increased the luciferase activity of the high Zn group; for the same *RREB1*-mutated group, the luciferase activity was significantly higher in the low Zn group than those in the control and the high Zn group (Figure 4A).

For *ZIP8* promoter, the mutation of the −1543/−1557 *MTF-1* binding site (8Mut-*MTF-1*-1), but not −483/−497 binding site (8Mut-*MTF-1*-2), eliminated the Zn-induced increase of promoter activity (Figure 4B).

### 2.4. EMSA (Electrophoretic Mobility Shift Assay) for the Confirmation of the Functional Binding of MTF-1 and RREB1 on the ZIP3 and ZIP8 Promoters

**Figure 5:**
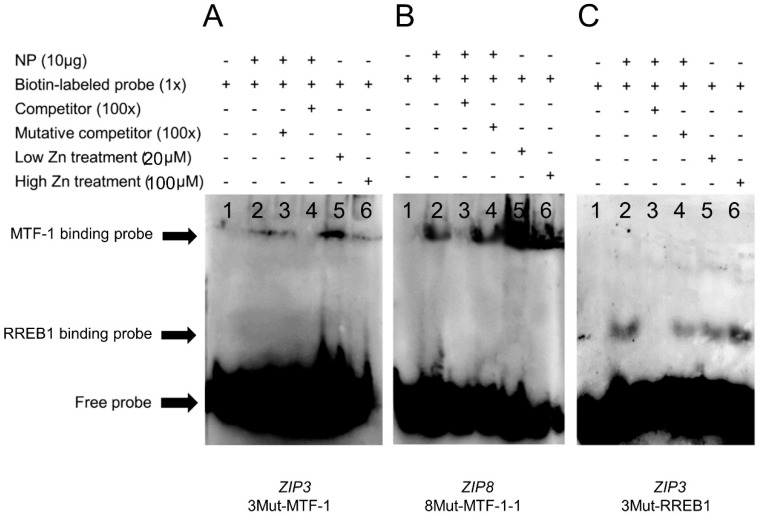
EMSA (electrophoretic mobility shift assay) of predicted *MTF-1* and *RREB1* binding sequences on the yellow catfish *ZIP3* and *ZIP8* promoters. (**A**) *MTF-1* binding sequences sited between −1327 bp and −1343 bp of *ZIP3* promoter; (**B**) *MTF-1* binding sequences sited between −1543 bp and −1557 bp of *ZIP8* promoter; (**C**) *RREB1* binding sequences sited between −248 bp and −267 bp of *ZIP3* promoter. NP, nuclear protein. The numbers 1–6 represent the six different lanes.

Based on the site mutation assays, the −1327 bp/−1343 bp for *MTF-1* binding and −248 bp/−267 bp for *RREB1* binding on the *ZIP3* promoter, and −1543 bp/−1557 bp for *MTF-1* bindings on the *ZIP8* promoter were considered to be functional. In order to confirm whether these sites can interact with *MTF-1* and *RREB1*, the EMSA was conducted. For the *ZIP3* promoter, when the *MTF-1* binding sequence was used as the probe, the 100-fold unlabeled *MTF-1* binding sites (−1327 bp/−1343 bp) competed for the binding, and the 100-fold unlabeled Mut-*MTF-1* binding sites reduced this competition, suggesting that *MTF-1* could bind with this region (Figure 5A). Meantime, results showed that low Zn promoted the binding of *MTF-1* to its corresponding binding site on the *ZIP3* promoter. When the *RREB1* binding sequence was used as the probe, the 100-fold unlabeled *RREB1* binding site (−248 bp/−267 bp) competed for the binding, and the 100-fold unlabeled Mut-*RREB1* binding region reduced this competition, suggesting that *RREB1* could bind with this region (Figure 5B). Additionally, compared with the band in the control, high Zn increased the brightness of the band, indicating that high Zn mediated the transcriptional regulation of *ZIP3* by *RREB1*.

For *ZIP8* promoter, when the *MTF-1* binding sequence was used as the probe, the 100-fold unlabeled *MTF-1* binding site (−1543 bp/−1557 bp) competed for the binding, and the 100-fold unlabeled Mut-*MTF-1* binding region reduced this competition, suggesting that *MTF-1* could bind this region (Figure 5C). Besides, Zn increased the brightness of bands compared with the bands in the control, indicating that Zn promoted the binding of *MTF-1* to its binding site on the *ZIP8* promoter.

## 3. Discussion

The investigation of the transcriptional regulation of Zn transporter proteins is an important aspect of Zn homeostasis in organisms. This study, for the first time, cloned and characterized the *ZIP3* and *ZIP8* promoters from a teleost fish, yellow catfish.

Studies suggested that the core promoter region was located most proximal to the start codon and contained the RNA polymerase binding sites [9]. In the present study, the core region of *ZIP* promoter had one TATA-box and one CAAT-box (NF-Y), similar to those in mouse *ZIP3* promoter (Ensembl ENSMUSG00000046822). TATA-box and CAAT-box are considered to dock the RNA polymerase transcriptional complex [10,11]. However, the core region of yellow catfish *ZIP* had neither TATA-box nor CAAT-box. Roy and Singer [9] reported that only about 5–7% of eukaryotic promoters had the TATA-box. Smale and Kadonaga [12] found that usually TATA-less promoters possessed Sp1 binding sites, which was also observed in the present study since two Sp1 binding sites were found in the core region of yellow catfish *ZIP8* promoter. Aiba et al. [13] pointed out that suppression of binding site Sp1 down-regulated *ZIP8*, suggesting the role of Sp1 in the control of *ZIP8* gene expression.

Identification of TFBS (transcription factor binding sites) contributes to decipher the mechanisms of gene regulation. The current study showed that the luciferase activities of the *ZIP3* promoter reduced when the sequence between −361 bp and −895 bp was deleted, suggesting that the positive regulator existed in this region. Further investigation found a cluster of binding sites, such as *PPARγ*, *STAT2*, *SREBP*, *MTF-1*, *RREB1*, *CREB*, *STAT2* and *KLF4* in the promoter region of *ZIP3*, reflecting that *ZIP3* participated in many physiological progress. For *ZIP8* promoter, some other binding sites, such as *STAT3*, *STAT4* and *CREB*, in the *ZIP* promoter region were found. This implies that the transcriptional regulation of *ZIP8* could be complicated and diverse transcription factors were involved in the regulation, and thereby play important roles in organisms. Moreover, the deletion of −897 bp/−1664 bp sequence reduced the luciferase activity, suggesting positive regulatory factors existed in this region. Similarly, one *MTF-1* binding site were found in this region. *MTF-1* was reported to positively regulate *ZIP* family [7,8].

Next, this study explored whether Zn incubation affected the activities of *ZIP3* and *ZIP8* promoters. The present studies indicated that for the *ZIP3* promoter, within the deletion plasmids of −2027 bp/+117 bp, the relative luciferase activities were the highest in the low Zn group and the lowest in the high Zn group, indicating that the regulation of *ZIP3* by Zn is Zn concentration-dependent. Similarly, several studies reported that *ZIP3* transcription and mRNA expression increased in responses to Zn deficiency [14,15], but reduced as Zn concentration increased [16]. In our laboratory, Chen et al. [5] found Zn addition down-regulated mRNA levels of *ZIP3* compared to the control in the hepatocytes. These results are consistent with the reported functions of these proteins in maintaining the homeostasis of Zn. Further site mutagenesis and EMSA identified a functional binding site of *MTF-1* in these regions. *MTF-1* functions as a cellular Zn sensor and binds specifically to MREs (metal response elements) to activate the expression of genes encoding Zn transporters in response to Zn ions [8,17,18]. Ling et al. [19] reported that 0.18 mg/L of waterborne Zn addition could significantly improve the mRNA expression level of *MTF-1* of juvenile goby *Synechogobius hasta*. Interestingly, EMSA showed that low zinc treatment had higher *MTF-1* binding activity than high zinc treatment (Figure 5A). One possible explanation was the presence of other transcriptional inhibitors in the promoter region of *ZIP3*, which can be activated by high zinc concentrations. This research implied that low Zn positively impacted ZIP3 expression probably through the *MTF-1* binding site located between −1289 bp and −2027 bp. Meantime, this study predicted a *RREB1* binding site from −248 bp to −267 bp and indicated that the mutation of the −248/−267 bp *RREB1* binding site in *ZIP3* alleviated the high Zn-induced reduction of the relative luciferase activities of *ZIP3* promoter. Furthermore, EMSA showed that the −248 bp/−267 bp sequence was a functional binding site, and that high Zn concentration promoted the *RREB1* binding to this site. Similarly, Franklin et al. [20] reported the presence of the potential *RREB1* binding sites in the *ZIP3* promoter and found that *RREB1* was a positive regulator for *ZIP3* gene expression. *RREB1* is a transcription factor which could activate or inhibit the transcription of various target genes, depending on the cell type, promoter features and co-binding proteins [21,22,23,24] of gene expression. Thus, the −248 bp/−267 bp *RREB1* site may play important roles in high Zn-induced down-regulation of *ZIP3* promoter activity.

For the *ZIP8* promoter, this study indicated that within the plasmid of −1664 bp/+234 bp, the luciferase activities increased with increasing Zn concentrations. Generally speaking, increasing *ZIP8* expression will increase intracellular Zn concentration, as suggested by Kim et al. [25]. Results also showed that, when the sequence was deleted from −1664 bp to −897 bp, high Zn reduced the luciferase activities, indicating that this region had the binding sequence that regulated *ZIP8* transcription. Further EMSA indicated that the −1543 bp/−1557 bp *MTF-1* binding site could be bound by *MTF-1*, and that Zn promoted the binding of *MTF-1* to its binding site on the *ZIP8* promoter. The indispensable role of Zn-*MTF-1* axis in regulating Zn homeostasis has been mentioned in several reports [25,26], and that fish and mammalian cells could respond to Zn^2+^ by increasing the *MTF-1* binding [8,27]. Andrews [17] pointed out that the *MTF-1* binding to the MRE was dependent on Zn and was easily disrupted when Zn was depleted.

In conclusion, the promoter regions of yellow catfish *ZIP3* and *ZIP8* were identified, and the binding sites of *MTF-1* and *RREB1* in the promoter regions were characterized. This study provides the first direct evidence for the interaction between *MTF-1*, *RREB1* and *ZIP3* and *ZIP8* genes in fish, and accordingly elucidates an innovative mechanism by which Zn regulates the transcriptional activities of *ZIP3* and *ZIP8*. These relevant studies are very important for the evaluation of Zn nutrition and toxicity in vertebrates.

## 4. Materials and Methods

### 4.1. Animals and Reagents

Yellow catfish used for promoter cloning were from a local commercial farm (Wuhan, China). HEK293T cell lines were obtained from the Cell Resource Center of Huazhong Agricultural University. Lipofectamine 2000 were purchased from Invitrogen. Passive Lysis Buffer and Dual-Luciferase were from Promega. Dulbecco’s Modified Eagle’s medium (DMEM), 0.25% trypsin-EDTA and fetal bovine serum (FBS) were bought from Gibco company (ThermoFisher Scientific, Waltham, MA, USA). Other reagents were analytical ones from Shanghai Sinopharm Group Corporation (Shanghai, China). The protocols for all animal and cell experiments followed the ethical guidelines of Huazhong Agricultural University (HZAU) for the care and use of laboratory animals and cells, and were approved by that university’s Ethics Committee (identification code: Fish-2018-0721, Date: 21 July 2018).

### 4.2. Promoter Cloning and Plasmid Construction

According to the full-length ZIP3 and ZIP8 cDNA sequences obtained in the studies [5], RNA ligase-mediated rapid amplification of 5′ cDNA ends (RLM-5′RACE) method was used to identify their 5′ cDNA sequences and the transcription start sites (TSS). The promoter cloning followed the method described in Xu et al. [28]. Briefly, genomic DNA was extracted from the tail fins of yellow catfish via the commercial kit (Omega, Norcross, GA, USA). The different primers were designed to analyze the sites of the first introns of *ZIP3* and *ZIP8* (Table S1). The high-efficiency thermal asymmetric interlaced-PCR (hiTAIL-PCR) method was used to clone the promoter sequences by designing the specific primers with overlapping sequences (Table S1). The luciferase reporter constructs were producted via the purified PCR product and pGl3-Basic vectors (Promega, Fitchburg, WI, USA). The ClonExpress II One Step Cloning Kit (Vazyme, Piscataway, NJ, USA) was utilized to ligate the products. Based on the distances from their TSS, this study decided to name the plasmids as the pGl3-2027/+117 of *ZIP3* vector and pGl3-1664/+234 of *ZIP8* vector, respectively. With the Erase-a-Base system (Promega) using templates of pGl3-2024/+117 of *ZIP3* vector, plasmids pGl3-361/+117, pGl3-895/+117 and pGl3-1289/+117 of *ZIP3* vector were generated. Utilizing the pGl3-1664/+234 of *ZIP8* vector as a template, the pGl3-249/+234 and pGl3-897/+234 of *ZIP8* vectors were obtained, respectively. The primers for the plasmid construction are listed in Table S2.

### 4.3. Sequence Analysis

This research predicted the putative transcription factor binding sites (TFBS) by these online tools (http://www.genomatix.de/ and http://jaspar.genereg.net/). The reference binding sequences are shown in Table S3. The Clustal-W multiple alignment algorithm was used to assess the sequence alignments.

### 4.4. Plasmid Transfections and Assays of Luciferase Activities

The experiment transfected the plasmid into HEK293T cells and assayed the luciferase activities followed the methods of Xu et al. [28]. Briefly, HEK293T cells were cultured in DMEM medium + 10% FBS. They were placed in a SANYO incubator for the cell culture with 5% CO_2_ at 37 °C. All these reporter plasmids were used in the equimolar amounts. They were co-transfected with 20 ng pRL-TK as the control. After 4 h, the transfection medium was changed to DMEM with 10% FBS. HEK293T cells were incubated with three Zn concentrations, including the control (without extra Zn addition), low Zn (20 μM Zn^2+^) and high Zn (100 μM Zn^2+^), respectively. Zn was added in the form of ZnSO_4_. The incubation continued for 24 h. The Dual-Luciferase Reporter Assay System was used to determine the luciferase activity.

### 4.5. Site-Mutation Assays of MTF-1 and RREB1 Binding Sites on the ZIP3 and ZIP8 Promoters

To analyze the *MTF-1* and *RREB1* binding sites on *ZIP3* and *ZIP8* promoters in yellow catfish, QuickChange II Site-Directed Mutagenesis Kit (Vazyme, Jiangsu, China) was used to perform this site mutagenesis. The pGl3-*ZIP3*-2144 and pGl3-*ZIP8*-1940 were used as the templates. The primers for mutagenic analysis are shown in Table S4. These constructs were named the 3Mut-*MTF-1*, 3Mut-*RREB1*, 8Mut-*MTF-1*-1 and 8Mut-*MTF-1*-2, respectively. Then, the present study utilized the reagent Lipofectamine 2000 (Invitrogen, Carlsbad, CA, USA) to co-transfect the pRL-TK and constructs into HEK293T cells. After 4h transfection, the transfection medium was changed to DMEM (10% FBS) (Thermo Fisher Scientific, Wilmington, DE, USA). After 24 h incubation, HEK293T cells were collected and the luciferase activities were analyzed.

### 4.6. Analysis of the Functional Binding Sites of MTF-1 and RREB1 on the ZIP3 and ZIP8 Promoters Based on Electrophoretic Mobility-Shift Assay (EMSA)

This study isolated proteins from HEK293T cells and performed EMSA assays to analyze the functional binding sites of *MTF-1* and *RREB1* on the regions of *ZIP3* and *ZIP8* promoters, based on Xu et al. [28]. Cytoplasmic and nuclear extracts were obtained based on the methods of Read et al. [29] and determined the protein contents by the bicinchoninic acid assay (BCA) method. Then, according to the LightShift Chemiluminescent EMSA Kit (Invitrogen, Carlsbad, CA, USA), each oligonucleotide duplex of *MTF-1* and *RREB1* binding sites were incubated with 10 μg nuclear extracts. Before the biotin-labeled probe was added, each unlabeled probe was pre-incubated for 10 min. The biotin-labeled probe was added at room temperature and the reaction continued for 30 min. Then they were detected via the electrophoresis on 6% native polyacrylamide gels. This research performed competition analyses by using 100-fold unlabeled oligonucleotide duplex with or without the mutation. Table S5 showed these oligonucleotide sequences for EMSA.

### 4.7. Statistical Analysis

All the data were presented as mean ± standard error of mean (SEM). Before the statistical analysis, the Kolmogorov–Smirnov test was used to determine the normality of the distribution of all the data. The homogeneity of variances was tested by Bartlett’s test. Data were analyzed with one-way ANOVA, Duncan’s multiple comparison and Student’s *t*-test where appropriate. *p* < 0.05 means statistically significant differences within the treatments. SPSS 19.0 software (SPSS, Chicago, IL, USA) was used to perform the statistical analyses.

## Supplementary Materials

The following are available online at https://www.mdpi.com/1422-0067/21/17/6135/s1, Table S1: Primers used for the cloning of ZIP3 and ZIP8 promoters in yellow catfish, Table S2: Primers used for 5′-deletion plasmids construction of yellow catfish ZIP3 and ZIP8 promoters, Table S3: The reference binding site sequences of multiple transcription factors on the promoter regions, Table S4: Primers used for site-mutation analysis, Table S5: Primers used for electrophoretic mobility-shift assay.
