# Supplementary material for: Functional Analysis of Two Zinc (Zn) Transporters (ZIP3 and ZIP8) Promoters and Their Distinct Response to MTF1 and RREB1 in the Regulation of Zn Metabolism
Source: Int J Mol Sci. 2020 Aug 26;21(17):6135. doi: 10.3390/ijms21176135 (PMC7503416; doi:10.3390/ijms21176135)
Supplement: Supplementary file 1 [file ijms-21-06135-s001.doc]

**Table S1.** Primers used for the cloning of *ZIP3* and *ZIP8* promoters in yellow catfish

| Gene | Forward primer (5'-3') | Reverse primer (5'-3') | Step |
| --- | --- | --- | --- |
| *ZIP3* | ACGATGGACTCCAGAGC  GGCCGCVVNVNNNCCAA | CTCTCACAGGGATGAGAAT | hiTAIL-PCR1 |
|  | ACGATGGACTCCAGAG | AGCTACAACCATCTCCATG | hiTAIL-PCR2 |
|  | ACGATGGACTCCAGAG | CGTTATCTCCATCAGACTCG | hiTAIL-PCR3 |
| *ZIP8* | ACGATGGACTCCAGAGC  GGCCGCVVNVNNNCCAA | CAGGGTGATGGCAGAAATTG | hiTAIL-PCR1 |
|  | ACGATGGACTCCAGAG | AGGTAGGAGTATATGTCTG | hiTAIL-PCR2 |
|  | ACGATGGACTCCAGAG | GGGACGCTTAACGTTGGTA | hiTAIL-PCR3 |

**Table S2.** Primers used for 5'-deletion plasmids construction of yellow catfish ZIP3 and ZIP8 promoters

| Gene | Primers | Forward primer (5'-3') | Reverse primer (5'-3') |
| --- | --- | --- | --- |
| *ZIP3* | pGl3-478/+62 | ctatcgataggtaccgagctcACAGTGCAAATGACCAGAC | cagtaccggaatgccaagcttGCTTCCGTTTGTACAAGAACC |
|  | pGl3-1012/+62 | ctatcgataggtaccgagctcATGGGATGTTGGGACAGACAG | cagtaccggaatgccaagcttGCTTCCGTTTGTACAAGAACC |
|  | pGl3-1502/+62 | ctatcgataggtaccgagctcGTAGCCAAAATTCTTAGGTTGTA | cagtaccggaatgccaagcttGCTTCCGTTTGTACAAGAACC |
|  | pGl3-2144/+62 | ctatcgataggtaccgagctcCATCTATTTAAGTCATAATCCA | cagtaccggaatgccaagcttGCTTCCGTTTGTACAAGAACC |
| *ZIP8* | pGl3-525/+234 | ctatcgataggtaccgagctcGGATCGACTCTGTGAGGATCC | cagtaccggaatgccaagcttGAGGGAGATGGTTATATTGGT |
|  | pGl3-1173/+234 | ctatcgataggtaccgagctcGCGAATTTCTTAAACGTC | cagtaccggaatgccaagcttGAGGGAGATGGTTATATTGGT |
|  | pGl3-1940/+234 | ctatcgataggtaccgagctcCATTCAGGTAGGAGCAGGA | cagtaccggaatgccaagcttGAGGGAGATGGTTATATTGGT |

**Table S3.** The reference binding site sequences of multiple transcription factors on the promoter regions

| Name | Binding site sequences | Name | Binding site sequences |
| --- | --- | --- | --- |
| *CREB* | TGAGCT | *RREB1* | CCMCMAMMCAMC |
| *KLF4* | MCACACCCW | *SREBP1* | TCACCCA |
| *MTF-1* | TNTGCACACG | *STAT2* | RAAACAGAAA |
| *NF-Y* | CCAAT | *STAT3* | TTCTNGGAA |
| *PPARα* | TRGGTNARAGGTCA | *STAT4* | YTTCYRGGAAR |
| *PPARγ* | GGNAAAGG | TATA-box | TATAAA |
| *PPARδ* | RRGGTCAAAGGTCA |  |  |

**Table S4.** Primers used for site-mutation analysis

| Gene | Primers | Forward primer (5'-3') | Reverse primer (5'-3') |
| --- | --- | --- | --- |
| *ZIP3* | 3Mut-*MTF-1* | cttttttgggctgattttaTAGATGAAGAGTCATAATGAGGAACACA | aaaatcagcccaaaaaagtAGTAAAAAATATTATCATGGAGAGAGTTTTT |
|  | 3Mut-*RREB1* | atggaatattgaattgggtTCATAGATCTGTGGTGCCTGGG | cccaattcaatattccataTCCCACATCCCCAGGTCC |
| *ZIP8* | 8Mut-*MTF-1*-1 | gaacttgcgttcagccaacATGCGTCGTCACATTTGTCCT | ttggctgaacgcaagttccACAATTCTTTAGCACTGTGGAGATTG |
|  | 8Mut-*MTF-1*-2 | agagccaagggcgagagagGGGAGGGAGAGCTGCTTGTG | tctctcgcccttggctctgGAAGTATAACAAAGTCTGAGTTCTCTTTCG |

**Table S5.** Primers used for electrophoretic mobility-shift assay

| Primers |  | Forward primer (5'-3') | Reverse primer (5'-3') |
| --- | --- | --- | --- |
| *ZIP3-MTF-1* | Biotin-pro  be | Biotin-ACTTTTTTTTTCTTCTTTTA | Biotin-TAAAAGAAGAAAAAAAAAGT |
|  | Mutative-c  ompetitor | ACTTTTTTGGGCTGATTTTA | TAAAATCAGCCCAAAAAAGT |
| *ZIP3-RREB1* | Biotin-pro  be | Biotin-TGTAAGTGGGTGGGGGGAGT | Biotin-ACTCCCCCCACCCACTTACA |
|  | Mutative-c  ompetitor | TGTAAGCAGGTTATAAAAGT | ACTTTTATAACCTGCTTACA |
| *ZIP8-MTF-1-1* | Biotin-pro  be | Biotin-GGAAATTGCACTCGGAAAAC | Biotin-GTTTTCCGAGTGCAATTTCC |
|  | Mutative-c  ompetitor | GGAACTTGCGTTCAGCCAAC | GTTGGCTGAACGCAAGTTCC |
